# Three dimensional printing of calcium sulfate and mesoporous bioactive glass scaffolds for improving bone regeneration *in vitro* and *in vivo*

**DOI:** 10.1038/srep42556

**Published:** 2017-02-13

**Authors:** Xin Qi, Peng Pei, Min Zhu, Xiaoyu Du, Chen Xin, Shichang Zhao, Xiaolin Li, Yufang Zhu

**Affiliations:** 1 Department of Orthopedic Surgery, Shanghai Jiao Tong University Affiliated Sixth People’s Hospital, Shanghai, China; 2 School of Materials Science and Engineering, University of Shanghai for Science and Technology, 516 Jungong Road, Shanghai 200093, China

## Abstract

In the clinic, bone defects resulting from infections, trauma, surgical resection and genetic malformations remain a significant challenge. In the field of bone tissue engineering, three-dimensional (3D) scaffolds are promising for the treatment of bone defects. In this study, calcium sulfate hydrate (CSH)/mesoporous bioactive glass (MBG) scaffolds were successfully fabricated using a 3D printing technique, which had a regular and uniform square macroporous structure, high porosity and excellent apatite mineralization ability. Human bone marrow-derived mesenchymal stem cells (hBMSCs) were cultured on scaffolds to evaluate hBMSC attachment, proliferation and osteogenesis-related gene expression. Critical-sized rat calvarial defects were applied to investigate the effect of CSH/MBG scaffolds on bone regeneration *in vivo*. The *in vitro* results showed that CSH/MBG scaffolds stimulated the adhesion, proliferation, alkaline phosphatase (ALP) activity and osteogenesis-related gene expression of hBMSCs. *In vivo* results showed that CSH/MBG scaffolds could significantly enhance new bone formation in calvarial defects compared to CSH scaffolds. Thus 3D printed CSH/MBG scaffolds would be promising candidates for promoting bone regeneration.

Scaffolds are promising for treating bone defects in bone tissue engineering. Such scaffolds should be osteoconductive, degradable and bioactive, and also should have a complex three-dimensional (3D) interconnected porous network and strong mechanical loading properties^1^,^2^. The main conventional approaches to fabricating porous bone tissue engineering scaffolds are polyurethane foam templating, gas foaming and the use of porogens to create pores. However, scaffolds prepared by gas foaming are not strong enough for bone implantation^3^ and polyurethane foam templating method is more appropriate to fabricate metal or ceramic scaffolds^4^,^5^,^6^. Although porogen-based methods can produce porous scaffolds with higher mechanical strength, the pores are not always interconnected^7^. Moreover, it is not easy to precisely control the pore morphology, pore size and overall porosity of scaffolds using these conventional methods. For example, to get ceramic scaffolds, polyurethane foam templating should be removed by a calcining process, which may make scaffolds shrink^8^. Recently, 3D printing technique has been developed to be an ideal method to prepare porous scaffolds, which could precisely control the scaffold architectures through computer-assisted design (CAD)/computer-aided manufacturing (CAM) under mild conditions. To date, a variety of biomaterials have been 3D printed to form scaffolds with controllable morphology, pore size and porosity, such as bioactive glass, hydroxyapatite, tricalcium phosphate and polymer scaffolds^9^,^10^,^11^,^12^,^13^,^14^.

Calcium sulfate hydrate (CaSO_4_·0.5H_2_O, CSH) is transformed into calcium sulfate dehydrate (CaSO_4_·2H_2_O, CSD) by reacting with water, and this has been used to produce sulfate cement for bone augmentation, drug carriers, and bone graft substitutes^15^,^16^,^17^. In addition, CSH is much more economic than many other biomaterials such as hydroxyapatite, calcium silicate or bioactive glass. Importantly, the mechanical properties of CSH cement can be enhanced after curing at 37 °C in 100% relative humidity for a period of time^18^,^19^. However, the weak acidic environment, poor bioactivity and too-rapid resorption rate of CSH-based materials *in vivo* have limited their clinical application^20^,^21^,^22^. Consequently, more efforts have been made to fabricate CSH-based cements by combining with other bioactive materials to improve bioactivity, stabilize pH environment and decrease resorption rate, as well as to tailor setting time and inject ability. Huan *et al*. introduced tricalcium silicate into CSH to produce a novel bone cement with better self-setting properties and *in vitro* bioactivity^23^, while Chen *et al*. improved the physicochemical properties and osteogenic activity of CSH cement by the addition of dicalcium silicate^24^. Petruskevicius *et al*. suggested that merging CSH with a less-resorbable calcium phosphate would be better for human applications^25^. However, most CSH-based bone cements are still inadequate for bone tissue engineering applications due to failure to improve both physicochemical properties and bioactivity simultaneously. So it is still necessary to create better CSH-based cement scaffolds for bone regeneration.

Recent studies have demonstrated that mesoporous bioactive glass (MBG) exhibits excellent bone-forming bioactivity, degradation and drug delivery properties, owing to its high specific surface area, large pore volume and mesoporous structure^26^,^27^,^28^. And much effort has been made to investigate the potential applications of MBG in bone tissue engineering. For example, Wu *et al*. reported 3D printed MBG scaffolds with a controllable pore architecture, excellent mechanical strength and good mineralization ability could be an excellent candidate for bone regeneration^29^. Zhang *et al*. used 3D printing to fabricate strontium-containing MBG scaffolds for bone regeneration, which combined the advantages of good bone-forming bioactivity with controlled ion release, drug delivery and enhanced compressive strength^9^. In addition, bioactive glass has an alkalescent degradation product and relative slow resorption^30^, which could be complementary with CSH. Moreover, to date there are no reports on combining the cement chemistry with the 3D printing technique for bone regeneration. Therefore, the aim of this study is to fabricate 3D porous CSH/MBG cement scaffolds for bone tissue engineering by 3D printing. Firstly, the incorporation of MBG can greatly improve the biological properties of composite scaffolds. Secondly, the solidification of CSH can further enhance the mechanical properties of CSH/MBG scaffolds. In addition, using the 3D printing technique, we can precisely control the architecture of a scaffold and enhance its mechanical stability.

## Results

### Characterization of MBG powder and CSH/MBG scaffolds

**Figure 1:**
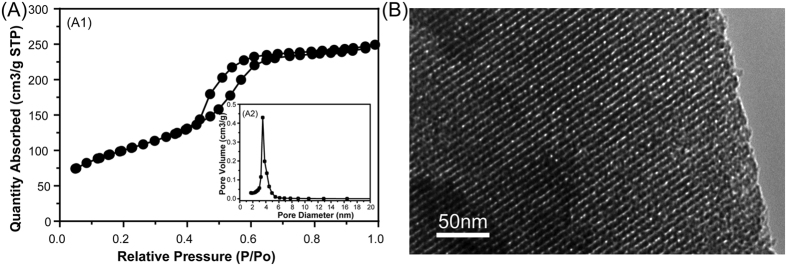
(**A**) N2 adsorption–desorption isotherms and the corresponding pore size distribution of MBG; (**B**) TEM image of MBG.

**Figure 2:**
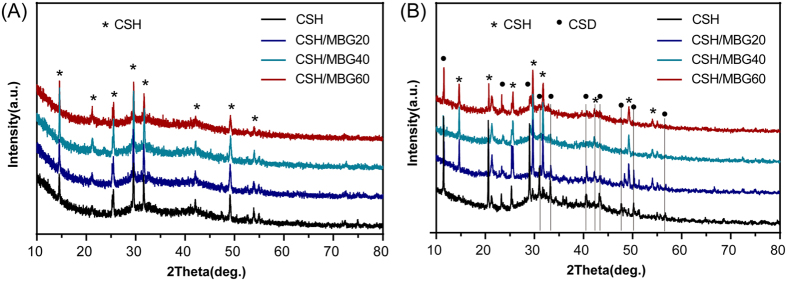
(**A**) XRD patterns of CSH and CSH/MBG scaffolds before and (**B**) after curing treatment for 3 days.

Figure 1A shows the N_2_ adsorption-desorption isotherm of MBG powder and the corresponding pore size distribution. The type IV isotherm with a hysteresis type H1 hysteresis loop (Fig. 1A1) was similar to that previously reported for mesoporous 58S bioactive glasses, revealing the P6 mm mesoporous structure of MBG powder^31^,^32^. The BET surface area and the single point total volume at P/P_0_ = 0.99 for MBG powder were 356 m^2^/g and 0.38 cm^3^/g, respectively. Figure 1A2 shows the pore size distribution curve of MBG, which was calculated from the desorption branches using the BJH model. The peak pore size was 3.94 nm. TEM observation showed that MBG powder contains highly ordered mesoporous channels (Fig. 1B), as previously reported for 58S bioactive glasses^31^. Figure 2 shows the XRD patterns of CSH/MBG scaffolds before and after incubation at 37 °C with 100% humidity for 3 days. Before incubation, peaks of CSH were observed in all samples (Fig. 2A), while after incubation peaks of CSD appeared in all the samples (Fig. 2B), indicating the incomplete hydration of CSH crystals following reaction with water. Because of the incomplete transformation of CSH to CSD, a hardening process occurred during incubation.

**Figure 3:**
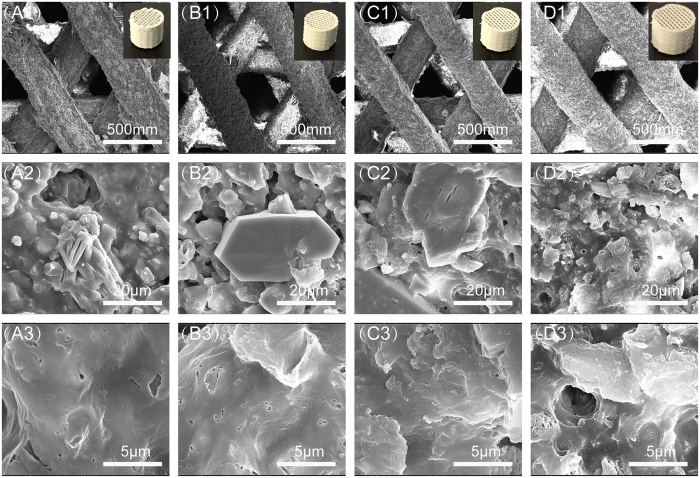
SEM images for the CSH (**A1**–**A3**), CSH/MBG20 (**B1**–**B3**), CSH/MBG40 (**C1**–**C3**) and CSH/MBG60 (**D1**–**D3**) scaffolds before soaked in SBF, 1 for ×200, 2 for ×5000 and 3 for × 20000; and inserted corresponding optical photographs printed by 3D Biopotter.

Photomicrographs and SEM images (Fig. 3) showed that all CSH/MBG scaffolds have the same architecture, with a regular macroporous structure. The parallel pore structure of fabricated scaffolds was quite uniform, and the pore size and strut diameter were ~350 μm and 400 μm, respectively. Magnified images of surfaces are shown in Fig. 3(A2–D3). MBG particles without regular morphologies are shown in CSH/MBG20, CSH/MBG40 and CSH/MBG60 specimens. However, rod-like CSD crystals were evident in CSH, CSH/MBG20 and CSH/MBG40 scaffolds, and the low content of CSD fillers in CSH/MBG60 scaffolds (Fig. 3D2–D3) induced a looser surface compared to the other three samples (Fig. 3A2–C3). In addition, it can be observed that many CSH crystals remained in the micropores formed by CSD stacking. The porosities of the CSH, CSH/MBG20, CSH/MBG40 and CSH/MBG60 scaffolds were estimated to be 66.7 ± 6.4%, 67.7 ± 5.4%, 68.2 ± 6.7%, and 68.3 ± 2.6%, respectively.

**Figure 4:**
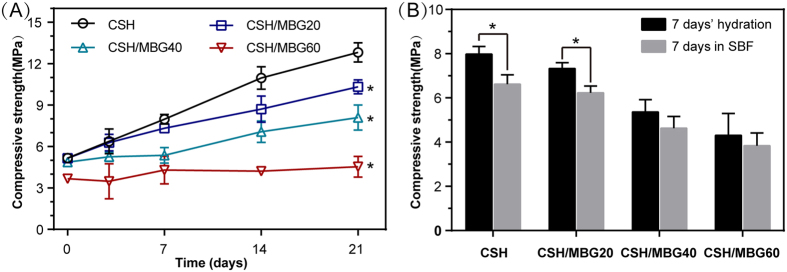
(**A**) Compressive strength of CSH and CSH/MBG scaffolds cured for various time periods (n = 3; *indicated significant differences compared to CSH, P < 0.05); (**B**) the compressive strength of CSH and CSH/MBG scaffolds after 7 days’ hydration and following 7 days’ immersion in SBF (n = 3; *indicated significant differences, P < 0.05).

The compressive strength of all 3D-printed scaffolds was measured after curing at 37 °C in 100% relative humidity for various time periods (Fig. 4A) and after SBF immersion for one week (Fig. 4B). With prolonging curing time, the compressive strengths of CSH, CSH/MBG20 and CSH/MBG40 scaffolds showed remarkable improvement. However, the compressive strength of CSH/MBG60 scaffolds exhibited no obvious change. After curing for 21 days, the compressive strengths of CSH, CSH/MBG20 CSH/MBG40, and CSH/MBG60 scaffolds were 12.8 ± 0.7 MPa, 10.4 ± 0.5 MPa, 8.2 ± 1.3 MPa and 4.5 ± 0.7 MPa, respectively. In addition, the compressive strength of scaffolds soaked in SBF for 7 days was slightly lower compared to those without SBF-soaked scaffolds (Fig. 4B).

### Degradation and apatite mineralization ability of CSH/MBG scaffolds

**Figure 5:**
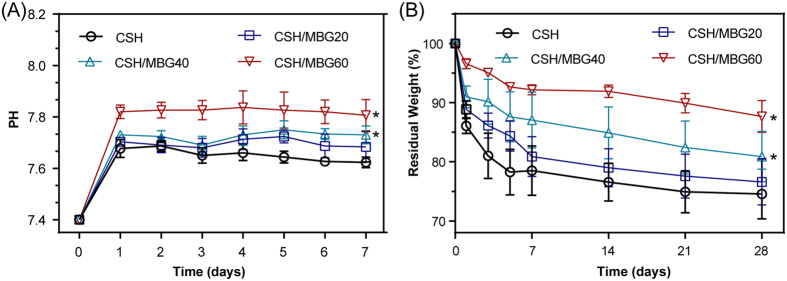
(**A**) PH values and (**B**) *in vitro* degradation of CSH/MBG scaffolds in SBF for various time periods (n = 3; *indicated significant differences compared to CSH, P < 0.05).

The variation of pH values as well as the weight losses of dried scaffolds were examined to check the degradation properties (Fig. 5). As shown in Fig. 5A, the pH values of the scaffolds changed following incubated in SBF solution. With increasing mass ratios of MBG, the pH of scaffolds-incubated SBF solution increased, which may contribute to ameliorating the inflammation caused by the acid microenvironment^19^. The pH values of various scaffolds immersed in SBF solutions for 7 days could be stabilized at 7.62, 7.68, 7.73 and 7.81 for CSH, CSH/MBG20, CSH/MBG40 and CSH/MBG60, respectively. In addition, as shown in Fig. 5B, the MBG addition could adjust the degradation rates of CSH/MBG scaffolds. Increasing MBG content caused the degradation rates of scaffolds to slow down, which could overcome the problem of overly rapid degradation of CSH^20^,^21^,^22^.

**Figure 6:**
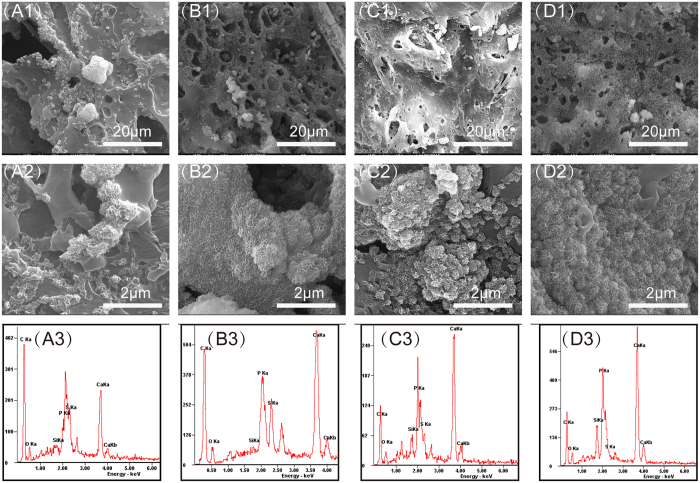
SEM images of the CSH (**A1**,**A2**), CSH/MBG20 (**B1**,**B2**), CSH/MBG40 (**C1**,**C2**) and CSH/MBG60 (**D1**,**D2**) scaffolds after immersed in SBF for 3 days. EDS analysis for the CSH (**A3**), CSH/MBG20 (**B3**), CSH/MBG40 (**C3**) and CSH/MBG60 (**D3**) scaffolds after immersed in SBF, respectively.

Surface morphologies of CSH/MBG scaffolds were characterized to evaluate apatite formation after 3 days of immersion in SBF, as shown in Fig. 6. Obvious differences in the hydroxyapatite layer could be observed on the surface of each scaffold compared to the relatively smooth surface before SBF immersion (Fig. 3). With increasing MBG content, the density of the apatite crystalline aggregates also increased. Besides, XRD analysis showed that hydroxyapatite peaks were detected on CSH/MBG scaffolds after soaked in SBF for 3 days (Fig. 1S). EDS analysis indicated the changes of the surface composition for all of CSH/MBG scaffolds after immersing in SBF, as shown in Fig. 6A3–D3. There are obvious characteristic peaks of Ca and P elements on EDS spectra after SBF immersion. The Ca/P ratios of CSH, CSH/MBG20, CSH/MBG40 and CSH/MBG60 scaffolds were 2.33, 1.89, 1.69 and 1.56, respectively. Because the apatite-layer of the CSH scaffolds was quite thin, Ca and S signals could also be detected, causing the over-high Ca/P ratio (2.33). However, for the other scaffolds, the Ca/P ratios were close to the 1.67 of hydroxyapatite.

### Cell responses to CSH/MBG scaffolds

**Figure 7:**
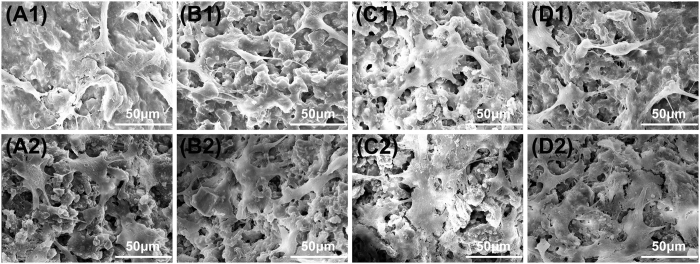
SEM images of the attachment of hBMSCs on the CSH (**A1**,**A2**), CSH/MBG20 (**B1**,**B2**), CSH/MBG40 (**C1**,**C2**) and CSH/MBG60 (**D1**,**D2**) scaffolds after cell culture for 3 days and 7 days, respectively.

We used hBMSCs to investigate cell response to CSH/MBG scaffolds. The attachment and morphology of hBMSCs on CSH/MBG scaffolds were observed by SEM (Fig. 7). After 3 days (A1–D1) or 7 days (A2–D2) of culture, hBMSCs were seen to be attached to the surface of the pore struts, exhibiting a well-spread morphology on each type of scaffold. However, the density of hBMSCs on the CSH/MBG20, CSH/MBG40 and CSH/MBG60 scaffolds was higher than that on the CSH scaffolds.

**Figure 8:**
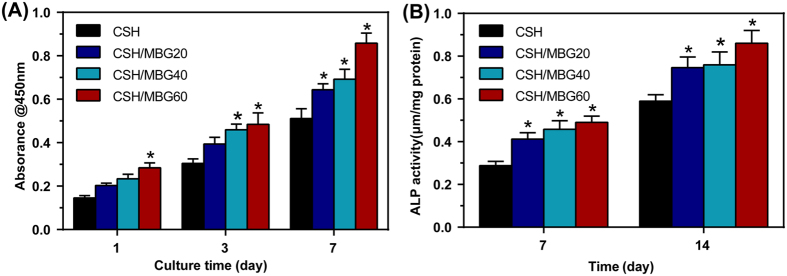
(**A**) Quantitative analysis of the proliferation of hBMSCs cultured on the CSH and CSH/MBG scaffolds shown for 1, 3 and 7 days (n = 3), (**B**) ALP activity of hBMSCs cultured for 7 and 14 days on the CSH and CSH/MBG scaffolds (^*^indicated significant differences when compared to CSH, *P* < 0.05).

The proliferation of hBMSCs cultured on the CSH, CSH/MBG20, CSH/MBG40 and CSH/MBG60 scaffolds for 3 and 7 days is shown in Fig. 8A. As determined by CCK-8 proliferation assay, the CSH, CSH/MBG20, CSH/MBG40 and CSH/MBG60 scaffolds all supported the proliferation of hBMSCs with increasing time in culture. However, the proliferation rates on the CSH/MBG20, CSH/MBG40 and CSH/MBG60 scaffolds were significantly higher than on the CSH scaffolds (*P* < 0.05). ALP activity of hBMSCs cultured on CSH/MBG scaffolds for 7 and 14 days are shown in Fig. 8B. Similar to the proliferation results, the CSH/MBG20, CSH/MBG40 and CSH/MBG60 scaffolds all exhibited significantly enhanced ALP activity compared to the CSH scaffolds (*P* < 0.05).

**Figure 9:**
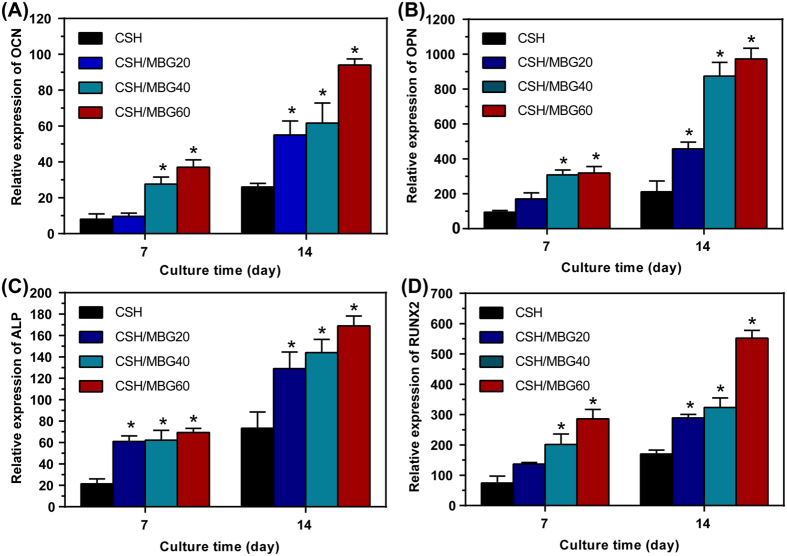
Osteogenic expression of OCN (**A**), OPN (**B**), ALP (**C**) and RUNX2 (**D**) for hBMSCs cultured on the CSH and CSH/MBG scaffolds by qRT-PCR analysis after 7 and 14 days (n = 3; ^*^indicated significant differences when compared to CSH, *P* < 0.05).

Cell differentiation of hBMSCs on CSH/MBG scaffolds was further evaluated by osteogenic expression determined by the expression of osteogenic markers OCN, OPN, ALP and RUNX2 at 7 and 14 days (Fig. 9). Results of gene expression analysis showed that the incorporation of MBG into CSH scaffolds could promote osteogenic differentiation of hBMSCs. With increasing culture time, expression of all the osteogenic-related genes was upregulated on CSH/MBG scaffolds. Moreover, the CSH/MBG20, CSH/MBG40 and CSH/MBG60 scaffolds exhibited enhanced expression levels compared to the CSH scaffolds (*P* < 0.05).

### Analysis of bone regeneration in calvarial defects

**Figure 10:**
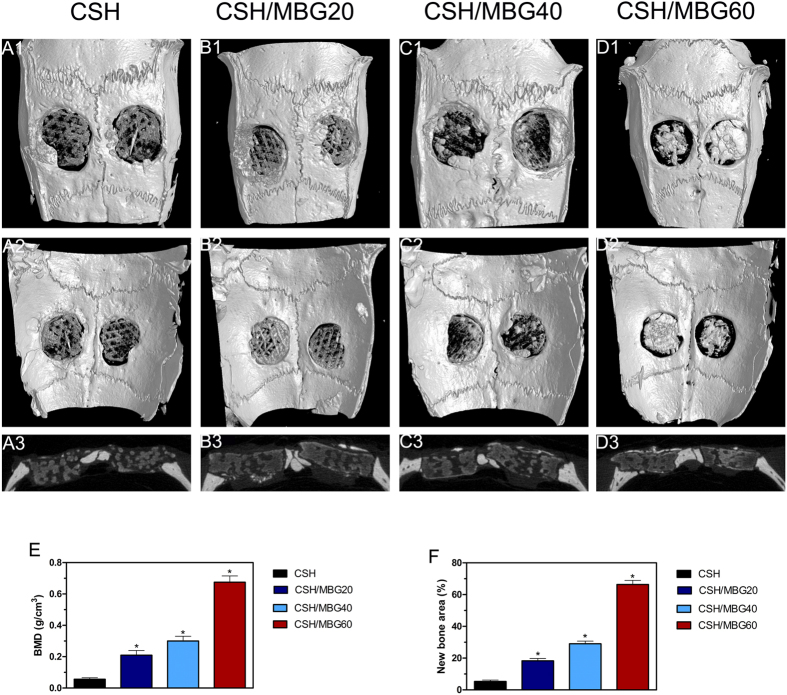
Micro-CT evaluation and morphometric analysis of calvarial defect bone repair. Representative 3D superficial (**A1**–**D1**), interior images (**A2**–**D2**) and sagittal images (**A3**–**D3**) of calvarial bone defects taken at 8 weeks after scaffold implantation. Morphometric analysis of bone mineral density (BMD) (**E**) and bone volume/total volume (BV/TV) (**F**) by micro-CT for each group at 8 weeks post-operation (^*^indicated significant differences when compared to CSH, *P* < 0.05).

Micro-CT images showing the 3D morphology and 2D slices of the repaired calvarial bones at week 8 are presented in Fig. 10. Figure 10A1–D2 depicts 3D morphological images of the newly-formed calvarial bones obtained using micro-CT reconstruction. In the sagittal view (Fig. 10A3–D3), little bone growth was observed in the defects in the CSH group. However, the CSH/MBG group showed increased new bone formation, attributable to the incorporation of MBG into CSH scaffolds. The local BMDs were 0.21 ± 0.03 g/cm^3^ in the CSH/MBG20 group, 0.3 ± 0.03 g/cm^3^ in the CSH/MBG40 group, and 0.675 ± 0.04 g/cm^3^ in the CSH/MBG60 group (Fig. 10E), and all of these were significantly different to the CSH group (0.056 ± 0.01 g/cm^3^) (*P* < 0.05). Moreover, BV/TV showed the same tendency as the BMD results (Fig. 10F), revealing a significant difference between the CSH/MBG20, CSH/MBG40, and CSH/MBG60 groups and the CSH group (*P* < 0.05). These results indicate that CSH/MBG scaffolds promote improved bone regeneration compared with CSH scaffolds, consistent with the results of qRT-PCR analysis.

**Figure 11:**
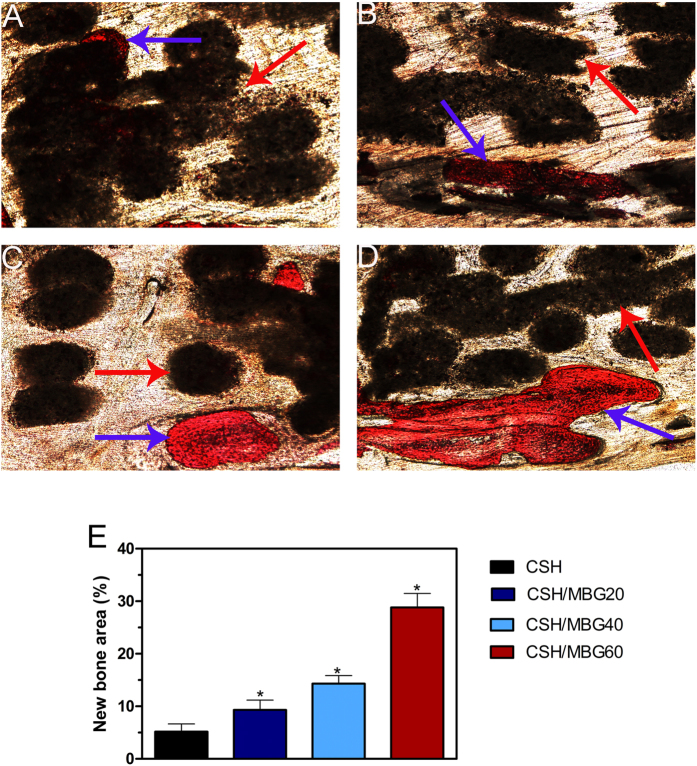
The un-decalcified crania were sectioned and stained with van Gieson’spicrofuchsin (×40). Representative histological photomicrographs of the newly-formed bone in the defect area (**A**–**D**), red arrow: CSH and CSH/MBG scaffolds, blue arrow: newly-formed bone. (**E**) The percentage of new bone area assessed at 8 weeks after implantation by histomorphometric analysis. (^*^indicated significant differences when compared to CSH scaffolds; *P* < 0.05).

Analysis of van Gieson’spicrofuchsin staining clearly showed that barely any new bone was formed in the CSH group (Fig. 11A), and only a small amount of new bone formation was observed in the CSH/MBG20 group (Fig. 11B). In contrast, in the CSH/MBG40 group (Fig. 11C), the ingrowth of newly-formed bone was evident in the central area of the defects as well as in the peripheral area near pre-existing bone. In the CSH/MBG60 group, bone formation was most active; the newly-formed bone tissues were relatively thick and almost covered the area of the defect (Fig. 11D). The histomorphometric results showed that the percentage of new bone area was significantly greater in the CSH/MBG20 scaffold group (9.33 ± 1.86%), the CSH/MBG40 group (14.33 ± 1.51%), and the CSH/MBG60 group (28.83 ± 2.64%) compared with the CSH group (5.17 ± 1.47%) (*P* < 0.05) (Fig. 11E).

## Discussion

In this study we successfully fabricated 3D porous CSH/MBG scaffolds using the 3D printing technique. The use of 3D printing allows precise control of pore size and pore morphology of the scaffolds as well as the struts. Polycaprolactone (PCL) has been approved as a biodegradable and biocompatible polymer by the US Food and Drug Administration (FDA), and has been widely used in clinical applications, such as prosthetic devices, implants, tissue-engineered skin and drug-delivery systems^9^,^12^,^13^,^33^,^34^. Therefore, PCL was used as the binder to fabricate CSH/MBG scaffolds. Compared with traditional CSH cements^18^,^23^, 3D printed CSH/MBG scaffolds had a regular and uniform square macropore structure, with pore size and porosity of approximately 350 μm and 68% (Fig. 2S), respectively. In general, a pore size of macropores greater than 150 μm and high porosity are ideal in scaffolds used for bone regeneration, as they facilitate cell proliferation, vascular ingrowth and internal mineralized bone formation^29^. Therefore, our 3D-printed CSH/MBG scaffolds had a desirable macroporous structure suitable for bone regeneration.

However, using polymers as binders to fabricate scaffolds for bone regeneration also has some drawbacks. For instance, the addition of polymers in printing paste as powder adhesives to enhance the strength of a scaffold, has proven to be inadequate^35^,^36^,^37^. In this study, however, various amounts of CSH crystals were incorporated into the CSH/MBG scaffolds. The rapid hydration and self-setting of CSH led to CSD solid formation in a relatively short period, hardening the ceramic powders and thus improving the initial scaffold strength. As illustrated in Fig. 3, the transformation of CSH rods into CSD sheets occurred after curing treatment, which decreased the porosity of the whole system and made particles compact. Furthermore, as shown in Fig. 4, the mechanical strengths of CSH/MBG scaffolds were remarkably improved by water treatment. Replacement of 20% of CSH with MBG induced an approximately 50% increase in strength after one week of hydration, and the increase became as high as 110% after three weeks. In addition, 40% replacement of CSH also conferred some improvement of the strength, and the enhancement increasedby10% and 40% after 1 week and 3 weeks of water treatment, respectively. However, for MBG substitution of CSH up to 60%, water incubation had very little influence on the mechanical strength of CSH/MBG60 scaffolds, which remained around 4 MPa.

CSH has acidic degradation products, a property which is not beneficial for cell viability and proliferation^19^. Additionally, the overly-rapid degradation of CSH is also incompatible with the formation of new bone^19^,^21^,^22^. Calcium species in MBG particles were loosely doped in the silica network and readily released outwards. Hydrolization of Ca^2+^ induced production of OH^−^ groups, thus increasing the substitution of MBG into CSH could stabilize the pH environment of scaffolds (Fig. 5A). After one week’s immersion in SBF, the pH of CSH/MBG60 scaffold was still maintained at 7.8. In addition, MBG has a slower degradation rate than CSH. So it is possible to tune the degradation rate of CSH/MBG scaffolds by changing the content of MBG, as shown in Fig. 5B.

The apatite layer formed on the surface of biomaterials in physiological fluid is very significant, contributing to osteoblastic activity, including proliferation and differentiation, and also predicting the *in vivo* bone bioactivity^28^,^31^,^38^. Our study shows that CSH scaffolds have very apatite mineralization. However, when the MBG content in CAH/MBG scaffolds was increased, the apatite layers on scaffolds were markedly increased (Fig. 6). The mechanism of apatite mineralization on CSH/MBG scaffolds is that the Ca^2+^ ions are first released from MBG powder to form a Si-rich layer, which then induces the formation of Ca–P nucleation and further apatite crystal formation^39^. Consequently, introducing MBG into calcium sulfate-based materials could improve their bioactivity.

The cell–material interaction has great influence on adhesion, motility, proliferation and differentiation of hBMSCs, which are the important steps that occur before bone mineralization^40^,^41^. CSH/MBG scaffolds increasingly stimulated the proliferation, ALP activity and osteogenesis-related gene expression of hBMSCs with the increasing the MBG addition. Compared to normal bioactive glass, MBG has higher Ca and Si contents and stable pH environment in cell culture medium, which may promote cell growth, proliferation and differentiation^42^. In this study we found that, compared to the CSH scaffolds, CSH/MBG scaffolds released Ca and Si ions and stabilized surrounding pH, creating a better microenvironment for osteogenesis, which may contribute to the enhanced adhesion, proliferation and differentiation of hBMSCs on CSH/MBG scaffolds.

The role of CSH/MBG scaffolds in bone regeneration *in vivo* was determined by testing their ability to repair critical-sized calvarial defects in a rat model. Micro-CT quantitative analysis showed that the CSH/MBG scaffolds could significantly improve osteogenesis in a calvarial defect model. CSH/MBG scaffolds significantly enhanced new bone formation, with the efficacy increasing with increasing content of MBG. Histological analysis also showed that there was little newly-formed bone in the defect areas in the CSH scaffold groups, whereas the CSH/MBG scaffold groups all significantly promoted bone formation, and the results were consistent with the micro-CT findings. The MBG addition in the CSH scaffolds could adjust the Si and Ca ions release, stabilize pH environment and match the degradation rate of CSH/MBG scaffolds with new bone formation, which contributed the better osteogensis capacity *in vivo* compared to CSH scaffolds.

## Conclusions

CSH/MBG scaffolds with hierarchical pore architecture were successfully prepared by the 3D printing technique. CSH/MBG scaffolds had uniform interconnected macropores, high porosity and improved mechanical properties. CSH/MBG scaffolds exhibited good apatite-forming ability. Moreover, our results showed that the addition of MBG into CSH scaffolds stimulated the adhesion, proliferation, ALP activity, and osteogenesis-related gene expression of hBMSCs. In *in vivo* studies, CSH/MBG scaffolds could significantly enhance new bone formation in calvarial defects compared to CSH scaffolds. Together these results suggest that 3D printed CSH/MBG scaffolds are promising candidates for promoting bone regeneration.

## Materials and Methods

Ethical approval for this investigation was obtained from the Research Ethics Committee of the Shanghai Sixth People’s Hospital-affiliated Shanghai Jiao Tong University. All methods were carried out in accordance with relevant guidelines and regulations of the Research Ethics Committee of the Shanghai Sixth People’s Hospital-affiliated Shanghai Jiao Tong University, all experimental protocols were approved by the Research Ethics Committee of the Shanghai Sixth People’s Hospital-affiliated Shanghai Jiao Tong University. Research carried out on humans must be in compliance with the Helsinki Declaration, human bone marrow-derived mesenchymal stem cells (hBMSCs) were obtained from three donors who gave their written informed consent.

### Materials

Nonionic block copolymer EO20PO70EO20 (P123, Mw = 5800) was purchased from BASF (Ludwigshafen, Germany). Hydrochloric acid (HCl, ≥36%), tetraethyl orthosilicate (TEOS, 98%), triethyl phosphate (TEP,99.8%), ethanol (99.7%) and calcium nitrate(Ca(NO_3_)_2_·4H_2_O, 99%) were purchased from Sinopharm Chemical Reagent Co. Ltd. (Shanghai, China) CaSiO_4_·1/2H_2_O (CSH, ≥97.0%) powders were purchased from Sigma-Aldrich (St Louis, MO, USA). Polycaprolactone (PCL, Mn70,000–90,000) was purchased from Sigma-Aldrich. All chemicals were used without further purification.

### Fabrication of CSH/MBG scaffolds by 3D printing

MBG powders (Si/Ca/P molar ratio 80/15/5) were prepared using nonionic block copolymer EO20PO70EO20 (P123) as the structure-directing agent, according to a previously-reported method^28^. 4th 3-D Bioplotter™ (EnvisionTEC GmbH, Germany) was used to fabricate 3D CSH/MBG scaffolds. Before printing the scaffolds, the injectable CSH/MBG paste was prepared as follows: First, different mass ratios (100:0, 80:20, 60:40, 40:60) of MBG and CSH powders were ground and passed through 300 mesh sieves, forming homogeneous powders with particle size of less than 45 μm. In this study, PCL was chosen as the binder for 3D printing. Next, 1.5 g of PCL was completely dissolved in 5 mL of chloroform, and 3.5 g of MBG/CSH powders mixture was added to the PCL solution, stirring quickly at room temperature to form an injectable paste. Finally, the prepared paste was introduced into a polyethylene injection cartridge which was fixed onto the 3D Bioplotter™ printer.

Simultaneously, cylinder models (φ8 × 2 mm, φ8 × 10 mm and their pore size was 350 μm) were loaded onto the Bioplotter CAD/CAM software and scaffolds were plotted layer by layer through the extrusion of the paste as a fiber, up to 25 layers. The architecture was changed by plotting fibers with 0 and 60 angle steps between two successive layers, the dosing pressure to the syringe pump was 2.2–3.6 bar and the speed of the dispensing unit was 4.5–8.2 mm/s, the nozzle size was 0.4 mm. Finally, the finished scaffolds were named as CSH, CSH/MBG20, CSH/MBG40 and CSH/MBG60, according to the different mass ratios of CSH to MBG powders. Before use, all scaffolds were cured in a 100% humidity water bath at 37 °C for various times.

### Characterization

Wide-angle XRD patterns were obtained on a Bruker D8 advance X-ray powder diffractometer (Bruker Corp., Billerica, MA, USA). Scanning electron microscopy (SEM) was carried out with an FEI Quanta 450 field emission scanning electron microscope (Thermo Fisher Scientific, Waltham, MA, USA). Transmission electron microscopy (TEM) was performed with a JEM-2010 electron microscope (Jeol Ltd., Tokyo, Japan) operated at an acceleration voltage of 200 kV. N_2_ adsorption–desorption isotherms were obtained on a MicromeriticsTristar 3020 (Micrometrics Instrument Corp., Norcross, GA, USA) at −196 °C under continuous adsorption conditions. Brunauer-Emmett-Tellwe (BET) and Barrett-Joyner-Halenda (BJH) methods were used to determine the surface area, pore size distribution and pore volume.

The compressive strength of CSH/MBG scaffolds (φ8 × 10 mm), stored in a water bath at 37 °C for different time-periods, was tested using a Zwick static materials testing machine (5kN) (Zwick Roell, Ulm, Germany) at a crosshead speed of 0.5 mm/min.

The porosity of CSH/MBG scaffolds was measured using Archimedes’principle: CSH/MBG scaffolds (φ8 × 10 mm) were used for the measurement and water was used as the liquid medium. The porosity (P) was calculated according to the following formula: P = (W_sat_ − W_dry_)/(W_sat_ − W_sus_) × 100%, where W_dry_ is the dry weight of CSH/MBG scaffolds, W_sus_ is the weight of CSH/MBG scaffolds suspended in water and W_sat_ is the weight of CSH/MBG scaffolds saturated with water.

### Degradation and apatite mineralization ability of CSH/MBG scaffolds

All the composite scaffolds were incubated in freshly-made simulated body fluid (SBF) at a ratio of 1 g scaffold per 200 mL SBF at 37 °C over a period of 30 days, with SBF changed every 7 days. The pH values of the solution and weights of dried scaffolds were recorded at each SBF change. SEM and energy dispersive spectrometry (EDS) were used to observe the surface morphology of all test scaffolds after soaking in SBF for 3 days.

### Cell response to CSH/MBG scaffolds

Briefly, marrow was extracted from the femoral mid shaft and then suspended in minimum essential medium containing 10% fetal bovine serum (Hyclone; GE Healthcare, Little Chalfont, UK), 100 U/mL penicillin and 100 mg/L streptomycin (Hyclone). Subsequently, the non-adherent cells were discarded; the adherent cells converged to 80–90% confluence and were then replaced as passage one (P1) cells. P3 cells were used for experiments.

### Cell adhesion and proliferation

hBMSCs (1 × 10^5^ cells/mL) were seeded onto sterilized scaffolds (5 mm in diameter ×3 mm) in 24-well culture plates and incubated in DMEM supplemented with 10% FBS at 37 °C in a humidified atmosphere of 5% CO_2_. After 3 and 7 days, the scaffolds with attached cells were removed, washed 3 times with PBS and fixed in 2.5% glutaraldehyde for 24 h. The fixed samples were washed 3 times with PBS and dehydrated through a graded series of ethanol (50%, 70%, 90%, 95%, and 100%) followed by soaking in hexamethyldisilizane (HMDS) for 4 h. The specimens were coated with gold and the morphological characteristics of the attached cells were examined using SEM (FEI Quanta 450).

The proliferation of hBMSCs on the scaffolds was assessed using a cell viability assay (Cell Counting Kit-8 (CCK-8); Dojindo Molecular Technologies, Inc., Kumamoto, Japan). Briefly, hBMSCs were cultured on the scaffolds (n = 3) following the procedure described above at an initial density of 1 × 10^4^ cells per scaffold for 1, 3 or7 days. At the end of the culture period, 360 μL of culture medium and 40 μL CCK-8 solution (9:1) were added to each well at each time point and the system was incubated at 37 °C for 4 h. Aliquots (100 μL) were removed from the wells and transferred to a fresh 96-well plate. The absorbance of the samples was measured at 450 nm with a spectrophotometric microplate reader (Bio-Rad 680; Bio-Rad, Hercules, CA, USA).

### Alkaline phosphatase (ALP) activity of hBMSCs on CSH/MBG scaffolds

To assess the osteoblastic differentiation of hBMSCs grown on the scaffolds, the ALP activity was measured on days 7 and 14 after 1 × 10^5^ hBMSCs were seeded onto each scaffold (n = 3). At the predetermined time-point, the culture medium was decanted, the cell layer was washed gently three times with PBS followed by washing once in cold 50 mM Tris buffer, and the hBMSCs were lysed in 200 μL 0.2% Triton X-100. Lysates were sonicated after being centrifuged at 14,000 g for 15 min at 4 °C, then 50 μL supernatant was mixed with 150 μL working solution according to the manufacturer’s protocol (BeyotimeInstitute of Biotechnology, Jiangsu, China). The conversion of p-nitrophenylphosphate into p-nitrophenol in the presence of ALP was determined by measuring the absorbance at 405 nm with a microplate reader (Bio-Rad 680).

### Osteogenic-related gene expression of hBMSCs on CSH/MBG scaffolds

The expression levels of the osteogenic-related genes runt-related transcription factor 2 (RUNX2), osteocalcin (OCN), alkaline phosphatase(ALP) and osteopontin (OPN) were measured using qRT-PCR. Typically, the cells were seeded at a density of 1 × 10^5^ cells per scaffold, cultured for 2 weeks and harvested using TRIzol Reagent (Invitrogen; Thermo Fisher Scientific) to extract the RNA. The obtained RNA was reverse-transcribed into complementary DNA (cDNA) using Revert-Aid First Strand cDNA Synthesis Kit (Thermo Fisher Scientific) and the qRT-PCR analysis was performed on an ABI Prism 7300 Thermal Cycler (Applied Biosystems, Foster City, CA, USA) using SYBR Green detection reagent. The relative expression of the genes of interest was normalized against the housekeeping gene β-actin. All samples were assayed in triplicate and independent experiments were performed. The relative expression was calculated using the following formula: 2^−(normalized average Ct)^ × 100.

### Animal experiments

Animal experiments were approved by the Research Ethics Committee of the Shanghai Sixth People’s Hospital-affiliated Shanghai Jiao Tong University, and performed in accordance with the Care and Use of Laboratory Animals protocols. Briefly, 48 mature male Sprague–Dawley (SD) rats (mean body weight 250–300 g) were provided with sterilized food and water and housed in a barrier facility with a 12-h light/dark cycle. These rats were randomly divided into four groups, each containing six rats. For the surgical procedure, as previously described^43^, the animals were anesthetized by intraperitoneal injection of chloral hydrate (4%; 9 mL/kg body weight) and all operations were performed under sterile conditions. A 1.5-cm sagittal incision was made in the scalp and the calvarium was exposed by blunt dissection. Two critical-sized calvarial defects with a bilateral diameter of 5 mm were created using a dental trephine, and the scaffolds were then implanted into the defects. Following the operation, the animals received intramuscular antibiotic injections, were allowed free access to food and water and were monitored daily for potential complications. Eight weeks after the operation, the rats were killed by an overdose of anesthetic and their craniums were harvested and fixed in a 4% paraformaldehyde solution buffered with 0.1 M phosphate solution (pH 7.2) overnight before further analysis.

### Microcomputed tomography (micro-CT) analysis

All the harvested specimens were examined using the mCT-80 system to evaluate new bone formation within the defect region. The undecalcified samples were scanned at a resolution of 18 μm. After 3D reconstruction, the bone mineral density (BMD) and bone volume fraction (bone volume/total volume [BV/TV]) in the defect regions were used to calculate new bone formation using the auxiliary software of the mCT-80 system^44^.

### Histological analysis

Each cranium was dehydrated through a graded alcohol series ranging from 70% to 100%, and then embedded in polymethylmethacrylate. After hardening, longitudinal sections were cut into 150–200 μm slices using a microtome (Leica Microsystems Ltd, Wetzlar, Germany), glued onto a plastic support and then polished to a final thickness of approximately 50 μm. New bone formation and mineralization were quantified at four locations that equally divided the defect site between the two ends of the longitudinal sections. The mean value of the four measurements was calculated to give average values for each group. The sections were then stained with van Gieson’s picrofuchsin to evaluate new bone formation^43^. The area of new bone formation was quantitatively evaluated at six random sections using Image Pro 5.0 software (Media Cybernetics, Rockville, MD, USA).

### Statistical analysis

The data were collected from three separate experiments and expressed as means ± standard deviation. The one-way ANOVA and Student–Newman–Keuls post hoc tests were used to determine the level of significance, and *P* values < 0.05 were considered to be significant.

## Additional Information

**How to cite this article**: Qi, X. *et al*. Three dimensional printing of calcium sulfate and mesoporous bioactive glass scaffolds for improving bone regeneration *in vitro* and *in vivo. Sci. Rep.* **7**, 42556; doi: 10.1038/srep42556 (2017).

**Publisher's note:** Springer Nature remains neutral with regard to jurisdictional claims in published maps and institutional affiliations.

## Supplementary Material

Supporting Information

Supplementary Figure 1S

Supplementary Figure 2S
