# Supplementary material for: Three dimensional printing of calcium sulfate and mesoporous bioactive glass scaffolds for improving bone regeneration in vitro and in vivo
Source: Sci Rep. 2017 Feb 13;7:42556. doi: 10.1038/srep42556 (PMC5304193; doi:10.1038/srep42556)
Supplement: Supporting Information [file srep42556-s1.pdf]

Title page

Title: Three dimensional printing of calcium sulfate and mesoporous bioactive glass scaffolds for improving bone regeneration in vitro and in vivo

Author list and affiliations:

Corresponding author: Min Zhu, Shichang Zhao,

Author list: Xin Qi[1,+], Peng Pei[2,+], Min Zhu[2,*], Xiaoyu Du[2], Chen Xin[2], Shichang Zhao[1,*], Xiaolin Li[1], Yufang Zhu[2]

Affiliations:

1 Department of Orthopedic Surgery, Shanghai Jiao Tong University Affiliated Sixth People's Hospital, Shanghai, China.

Xin Qi, Shichang Zhao, Xiaolin Li

2 School of Materials Science and Engineering, University of Shanghai for Science and Technology, 516 Jungong Road, Shanghai 200093, China.

Peng Pei, Min Zhu, Xiaoyu Du, Chen Xin, Yufang Zhu

[+]These authors contributed equally to this work.

Correspondence and requests for materials should be addressed to S.-C.Z. (e-mail: zhaoshichang0404@163.com) or M.Z. (e-mail: mzhu@usst.edu.cn)

Supporting information

1. The XRD analysis of CSH/MBG scaffolds after apatite mineralization.

All the composite scaffolds were soaked in a freshly made simulated body fluid (SBF) at a 1g scaffold per 200 ml SBF ratio at 37°C for 3 days. After soaked, the apatite peaks appeared but to some degree weak because of the insufficient soaking process.

Compared to Fig.2B, the peak intensity of CSH and CSD was decreased due to the quick degradation of CSH materials.

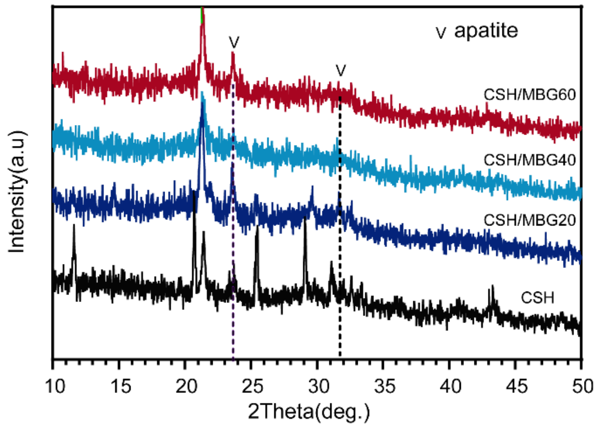

Fig.1S: the XRD analysis of CSH and CSH/MBG scaffolds after soaked in SBF for 3 days.

2. The porosity of CSH/MBG scaffolds.

The porosity of CSH/MBG scaffolds was measured using Archimedes'principle: CSH/MBG scaffolds (φ8 ×10mm) were used for the measurement and water was used as the liquid medium. The porosity (P) was calculated according to the following formula: $P = (W_{sat} - W_{dry}) / (W_{sat} - W_{sus}) \times 100\%$, where W_{dry} is the dry weight of CSH/MBG scaffolds, W_{sus} is the weight of CSH/MBG scaffolds suspended in water and W_{sat} is the weight of CSH/MBG scaffolds saturated with water.

Fig.2S showed the porosity of CSH, CSH/MBG20, CSH/MBG40 and CSH/MBG60 scaffolds were 67 ±1.17%, 67.63 ± 2.43%, 68.02 ±1.64% and 67.75 ±3.27%, respectively. The similar porosity of four group scaffolds indicated that 3D printing technique could precisely control the inner-structure of scaffolds.

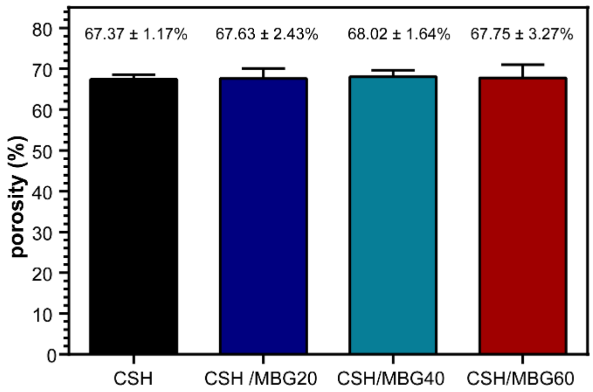

Fig.2S: the porosity of CSH/MBG scaffolds
