# Supplementary figures and images for: Three dimensional printing of calcium sulfate and mesoporous bioactive glass scaffolds for improving bone regeneration in vitro and in vivo
Source: Sci Rep. 2017 Feb 13;7:42556. doi: 10.1038/srep42556 (PMC5304193; doi:10.1038/srep42556)

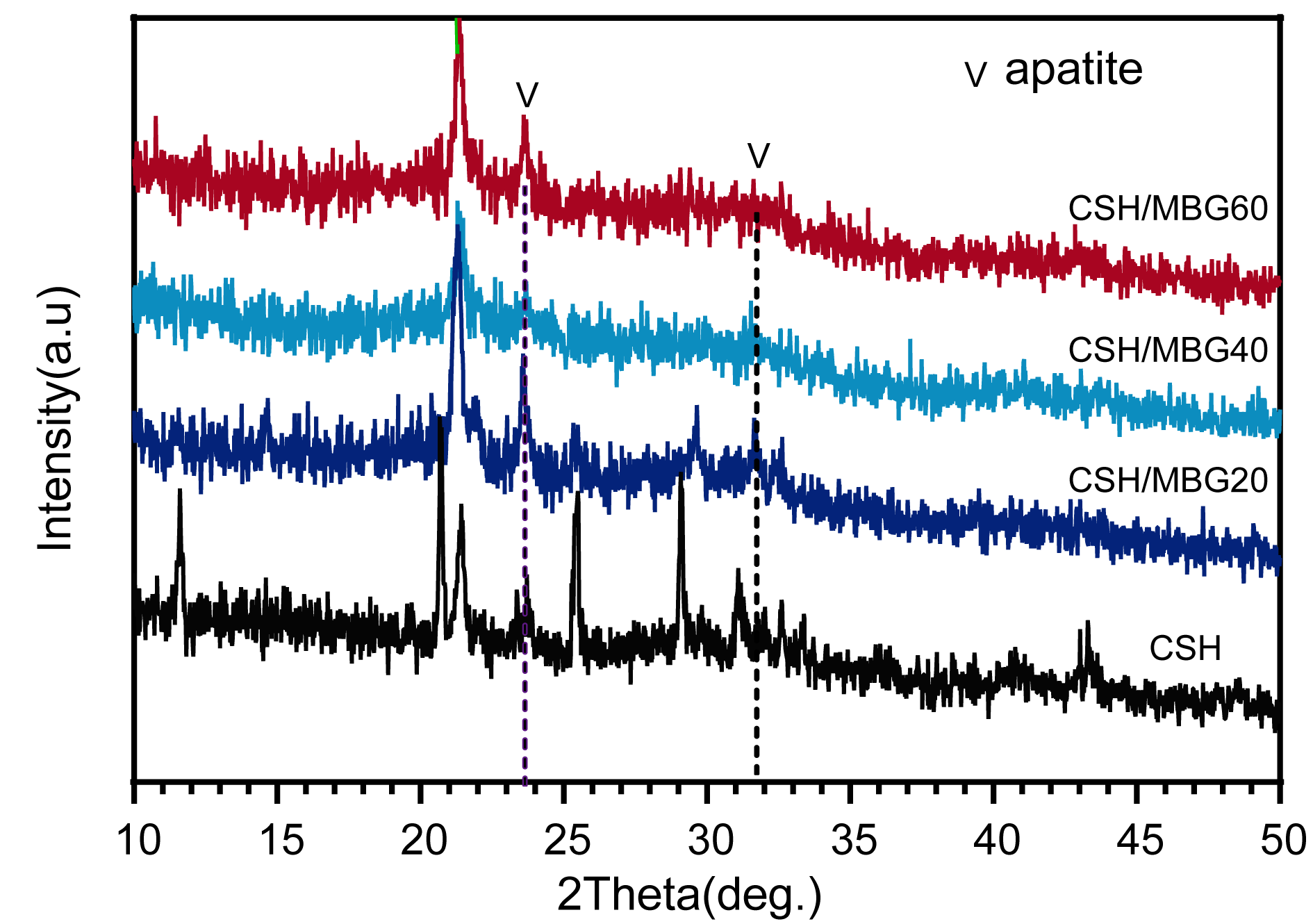

Supplement: Supplementary Figure 1S [file srep42556-s2.tiff]

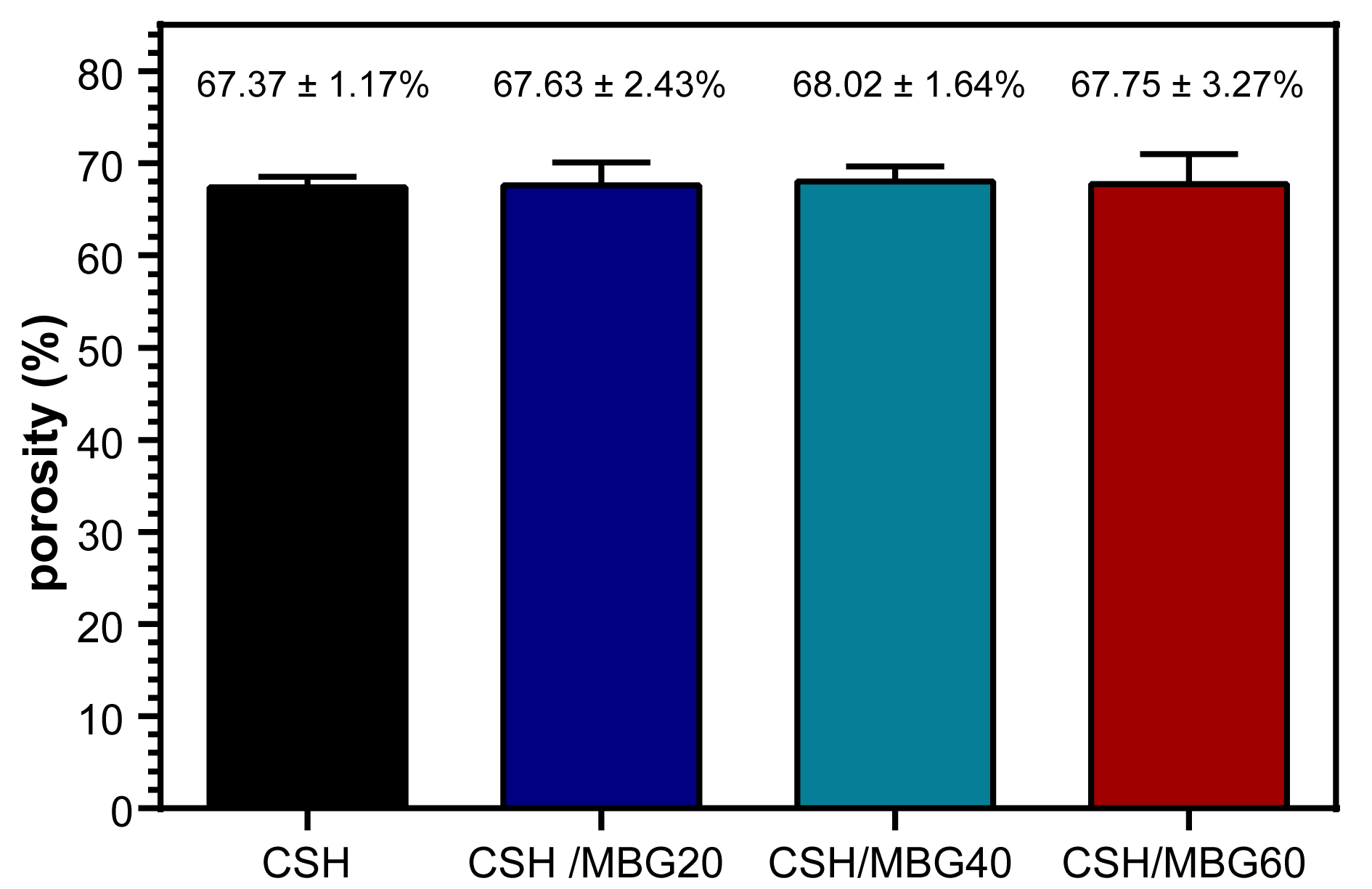

Supplement: Supplementary Figure 2S [file srep42556-s3.tiff]
